# Dense Percolation in Large-Scale Mean-Field Random Networks Is Provably “Explosive”

**DOI:** 10.1371/journal.pone.0051883

**Published:** 2012-12-18

**Authors:** Alexander Veremyev, Vladimir Boginski, Pavlo A. Krokhmal, David E. Jeffcoat

**Affiliations:** 1 Department of Industrial and Systems Engineering, University of Florida, Gainesville, Florida, United States of America; 2 Research and Engineering Education Facility (REEF), University of Florida, Shalimar, Florida, United States of America; 3 Department of Mechanical and Industrial Engineering, University of Iowa, Iowa City, Iowa, United States of America; Universitat Rovira i Virgili, Spain

## Abstract

Recent reports suggest that evolving large-scale networks exhibit “explosive percolation”: a large fraction of nodes suddenly becomes connected when sufficiently many links have formed in a network. This phase transition has been shown to be *continuous (second-order)* for most random network formation processes, including classical mean-field random networks and their modifications. We study a related yet different phenomenon referred to as *dense percolation*, which occurs when a network is already connected, but a large group of nodes must be *dense enough*, i.e., have at least a certain minimum required percentage of possible links, to form a “highly connected” cluster. Such clusters have been considered in various contexts, including the recently introduced *network modularity principle* in biological networks. We prove that, contrary to the traditionally defined percolation transition, dense percolation transition is *discontinuous (first-order)* under the classical mean-field network formation process (with no modifications); therefore, there is not only quantitative, but also qualitative difference between regular and dense percolation transitions. Moreover, the size of the largest dense (highly connected) cluster in a mean-field random network is explicitly characterized by rigorously proven tight asymptotic bounds, which turn out to naturally extend the previously derived formula for the size of the largest clique (a cluster with all possible links) in such a network. We also briefly discuss possible implications of the obtained mathematical results on studying first-order phase transitions in real-world linked systems.

## Introduction

In recent years, “explosive percolation” in large-scale random networks has received substantial attention. This phenomenon essentially means that as new links are randomly added step-by-step to a network with 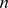 nodes (assuming a very large 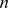) and the number (or percentage) of links reaches a certain critical threshold (the phase transition point), the order of magnitude of the largest connected component changes abruptly from logarithmic in 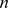 to linear in 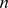, which is often referred to as the emergence of a giant connected component.

Achlioptas et al. [1] recently reported an interesting computational study suggesting that although the percolation transition in the classical Erdös-Rényi (mean-field) random graph model (e.g., links being added to the network independently and with the same probability) is continuous, this transition can be made discontinuous (or, “explosive”) by applying modified network formation rules referred to as Achlioptas processes. Several related studies also addressed explosive percolation in various models [2]–[6]. However, other studies showed that explosive percolation is still continuous in most settings [7]–[9]. Recently, Riordan and Warnke [10], [11] proved that explosive percolation is continuous for all Achlioptas processes. As follows from these recent studies, the current state of knowledge on the classical percolation transition phenomena is that these phase transitions are continuous (*second-order*) under most network formation rules, but they can be discontinuous (*first-order*) if a network formation process is substantially different from the Erdös-Rényi model.

However, if one looks at the percolation transition phenomenon from the perspective of system *robustness*, percolation by itself does not necessarily ensure that a connected network, which has just undergone a percolation transition, is stable and resilient with respect to natural or man-made (possibly adversarial) impacts, which may disrupt multiple links and violate the overall integrity of the underlying system. In other words, the connectivity of the whole network or a large fraction of nodes (giant connected component) may not be sufficient for maintaining the “stability” of the linked system. For instance, intuitively, consider a group of particles linked by bonds: if the *relative number* of bonds is too small, this connected group of nodes would not form a stable structure (i.e., a crystal), since the destruction of a just a few bonds may disconnect the system. Note that a linear in 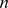 number of links (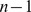 being the exact minimum) is sufficient to fully connect a system of 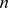 nodes. On the contrary, it is much harder to disconnect a connected cluster containing close to the maximum possible number of links (i.e., 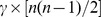 links, where 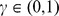 is sufficiently close to 1). Such “dense” connected clusters will be a subject of special interest further in this paper.

“Dense” (or, “highly connected”) network clusters have recently received attention in diverse research areas, including those dealing with biological, social, communication, and financial networks, and, as it will be discussed below, these clusters may correspond to “functional units”, or “modules” in an underlying complex system, which is reflected in the so-called *network modularity* principle [12], [13]. However, it turns out that finding large dense connected clusters in a network is a much more computationally challenging task than identifying “regular” connected components. Moreover, despite many previous results on the properties of connected components in random networks, dense clusters have not been studied nearly as much as “regular” connected components.

Therefore, in this work, we pursued a line of thought deviating from most of the recent percolation studies in random networks: instead of concentrating on various network formation rules (i.e., Erdös-Rényi vs. Achlioptas processes) and analyzing the traditional percolation transition phenomenon with the emerging giant connected component, we considered a different type of phase transition with the emphasis not just on connectivity, but on *dense connectivity*. We then rigorously analyzed how the *dense percolation transition* occurs under the classical Erdös-Rényi (mean-field) network evolution process, as well as how the largest dense cluster size behaves when the whole network is sparse.

## Results

### Dense Clusters in Networks

**Figure 1:**
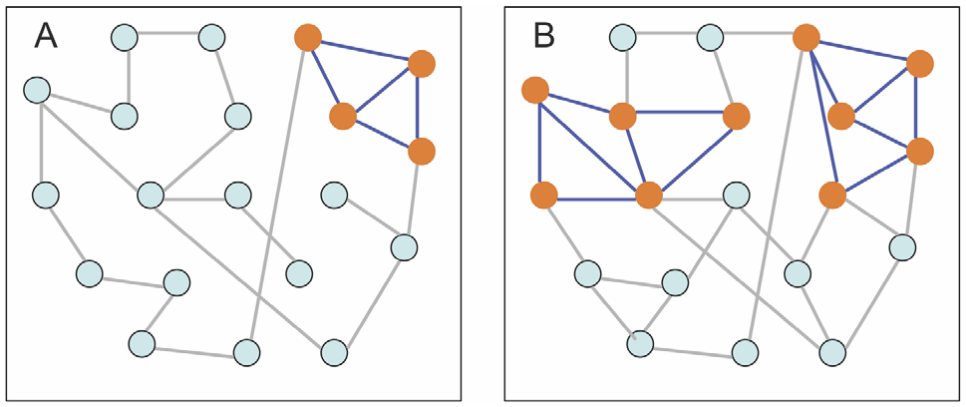
Small-scale illustration of the concept of dense connected clusters in comparison with traditionally defined connected components. (**A**) A connected network with 20 nodes and 22 links. Assuming that the minimum required percentage of links for a group of nodes to form a dense connected (“highly connected”) cluster is 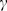 = 70% (this value is chosen arbitrarily for illustrative purposes only), the largest-size dense cluster is 4, and all other dense clusters have only 2 nodes each. (**B**) The network from (A) after 10 more links have formed (for instance, this may be the result of increasing the value of 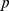 if one assumes 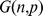 model). Dense clusters with the largest number of nodes are highlighted, with their size still significantly smaller than the size of the whole network. It turns out that if 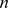 is very large, further increase of 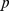 in 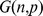 will only produce dense clusters scaling as 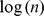 (no clusters scaling linearly with 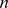) until 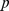 reaches the critical point 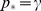, after which the whole network abruptly becomes a dense cluster.

To facilitate the description of the main results, we first introduce basic definitions and briefly discuss the relationship of dense connected clusters and “regular” connected components in networks. A *dense connected component* (cluster) in a network is defined in terms of its *edge density* parameter 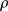, which is equal to the ratio of the actual number of links (edges) to the maximum possible number of links within the cluster (in other words, the relative percentage of links within the cluster – see Fig. 1 for illustration). In the basic graph-theoretic model, the minimum required edge density for a cluster to be dense enough (“stable”) does not depend on the size of the considered cluster and is set to a fixed parameter 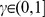, which may be chosen depending on application-specific considerations (that is, a cluster is considered dense enough, if 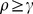). Note that although the minimum required edge density 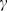 is fixed, the number of links required to form a dense cluster on 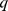 nodes does depend on the size of the cluster 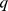 and is equal to 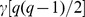. The concept of dense clusters was also recently addressed in the context of *network modularity* principle described by Hartwell et al. [12] and further analyzed by Spirin and Mirny [13], who considered “*highly connected subgraphs (clusters)*” (or, “modules”) defined using the aforementioned edge density parameter, which represented meaningful functional units in molecular networks. The significance of dense clusters in other research areas will be addressed in the next section.

In graph theory, dense (“highly connected”) clusters described above are referred to as 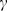 *-quasi-cliques* [14], [15], with the limiting case of 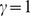 corresponding to the well-known *clique* structure, where there is a link between *every* two nodes. Clearly, cliques are very cohesive and resilient to node and link failures; however, large-scale real-world systems rarely form cliques, as it is often unrealistic to connect every two nodes by a link. Therefore, quasi-cliques provide a reasonable tradeoff between regular connected components, which do not necessarily reflect network modularity and may be “not robust enough” (as indicated above), and cliques, which are “too robust”. The assumption that the required threshold value of 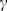 for a cluster with 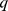 nodes to form a quasi-clique (“stable”/“highly connected” cluster) is constant and does not depend on 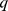 may not always be fully realistic; however, it does make theoretical and practical sense, since if one considers the large-scale (asymptotic) case with 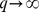, the function 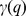 should not vanish as 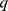 increases, since otherwise this would represent an arbitrarily low edge density of a cluster, which would effectively “downgrade” a dense connected cluster to a regular connected component.

In related work, the concept of a 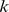-core (a connected subgraph where each node has a degree of at least 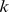, that is, at least 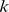 neighbors) has been analyzed in mean-field random networks [16]–[18], and D’Souza [19] discussed the notion of “dense 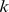-cores” as clusters that may possess extra robustness properties in addition to connectivity. However, if 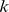 is fixed and 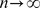 there exist 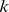-cores with edge density arbitrarily close to zero. Therefore, a more meaningful type of a “robust cluster”, which preserves the required edge density in addition to the minimum required degree of each node, is a slightly modified version of a 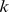-core, where 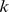 is defined in terms of the *percentage* of all possible neighbors within a cluster, i.e., 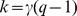 [20]–[22], that is, the number of neighbors for each node within the cluster *depends on the size of this cluster*. It is easy to observe that any 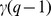-core is also a 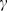-quasi-clique, but the reverse is not always true. Therefore, 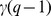-cores represent a slightly more restrictive type of “highly connected” clusters than 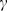-quasi-cliques. In addition, 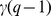-cores possess certain guaranteed robustness properties if 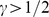: as it straightforwardly follows from [23], a 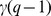-core of size 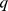 with 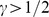 has a diameter of at most 2 (that is, any two nodes are connected through at most one intermediary), and it would stay connected after the removal of up to 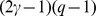 links.

Under the aforementioned assumption of the fixed required value of 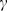, we posed a natural question: how does the size of the largest dense connected cluster (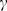-quasi-clique or 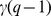-core) grow as more and more links are added to an evolving large-scale network? In particular, can one rigorously prove the emergence of a “giant dense connected component” and investigate the order of this phase transition (i.e., whether it is continuous or discontinuous)? It turns out that in the context of the classical 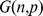 (Erdös-Rényi) model, these questions can be answered unambiguously.

### First-Order Dense Percolation Transition in Erdös-Rényi Networks

Formally, an Erdös-Rényi random graph 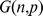 contains 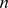 nodes, and each pair of nodes is connected by a link independently with probability 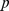. The process of evolution of such a network can be represented simply by a gradual increase of the parameter 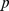, with larger values of 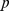 representing more links in the network. Although this is a somewhat idealized classical model, the issues of its appropriateness in certain contexts will be addressed in the next section.

One may intuitively assume that the size of the largest dense connected component in 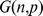 would first grow logarithmically (while 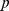 is much smaller than 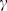) and then linearly right after some critical point 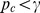, and it would continue to grow until it eventually reaches 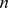, similarly to the asymptotic behavior of the giant connected component in an Erdös-Rényi network; however, our rigorous mathematical arguments show that this is not the case. As our results show, for 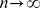, the largest dense connected cluster scales as 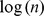 as long as 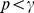 (even if 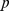 is very close to 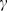), whereas when 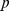 reaches 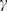, *the whole network* abruptly becomes a dense connected cluster. Essentially, for very large values of 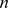, the jump from the logarithmic order of magnitude of the largest dense cluster to the largest dense cluster of size 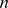 occurs at *one point* 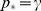, with no “linear growth” phase in between! Therefore, for any fixed 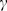, there is no emerging giant dense connected component as 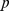 gradually increases, until a discontinuous jump at the point 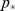 produces a dense connected component representing the entire network. In other words, the size of the largest dense connected component in a large-scale Erdös-Rényi random network exhibits a *first-order phase transition*; moreover, the existence of this phase transition has been proven by fully rigorous analytical arguments.

The complete detailed proofs are presented in the *Materials and Methods* section, and here we briefly summarize the obtained formal results. The following proven facts characterize the size of the largest dense connected component (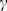-quasi-clique) in 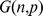 for sufficiently large 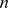.

If 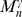 is the size of the maximum 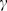-quasi-clique in 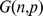 for some fixed 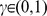, then for any 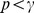 it holds *almost surely* (a.s.) that
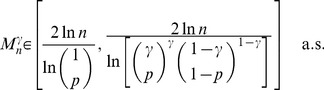
(1)


In this context, a property 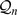 is said to hold *almost surely* (a.s.), if with probability 1 there exists 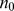 such that 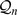 holds for all 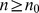.

Formula (1) is also valid for the size of the *largest 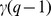-core* (in the context of the above description) in 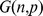. Moreover, *with high probability* (w.h.p.)
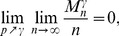
(2a)but
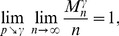
(2b)where a property 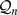 is said to be observed with high probability (w.h.p.) if the probability of 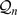 being observed converges to 1 as 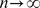.

Expression (1) entails that the size of the largest 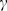-quasi-clique does scale logarithmically with 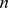 for any 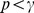. Note that it provides not only the order of magnitude, but also asymptotically precise upper and lower bounds on the size of the largest 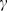-quasi-clique. An interesting observation here is the fact that the upper bound converges to the lower bound as 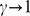, whereby (1) becomes the classical asymptotic estimate for the maximum clique size in 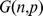 described by Bollobás and Erdös [24] and Grimmett and McDiarmid [25].

Formulas (2a) and (2b) formally express the existence of the first-order (discontinuous) jump of the size of the largest dense connected component (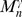) compared to the size of the whole network (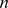): if 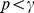 and 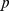 approaches 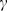 from below (that is, the edge density of the whole network is just below the required edge density 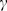), the size of the largest cluster that does have the required density 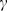 is still negligible compared to the size of the whole network. For 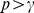 and approaching 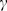 from above, the whole network forms the dense cluster, and as mentioned above, there is no “gradual” (continuous) change in terms of the size of the largest dense cluster.

As a final remark, the fact that the location of the dense percolation transition is in the point 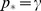 is not surprising and can be intuitively understood, since one would expect that the size of the largest dense connected cluster in 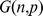 would be equal to 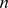 if 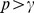 and less than 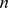 if 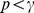. However, the fact that this phase transition is *first-order in the asymptotic case* is not intuitively predictable. This fact has been established via advanced analytical arguments that will be presented below.

## Discussion

In this section, we briefly discuss potential implications of the obtained results on studying network clusters and phase transitions in related research areas. Dense connected clusters (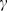-quasi-cliques, 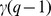-cores and similar models) have been recently utilized to study large-scale networked systems in a variety of disciplines, including biological networks [12], [13], [21], [26]–[29], social networks [30]–[33], telecommunication networks [14], [15], and financial networks [34]–[37]. On the other hand, the mechanisms of phase transitions in large-scale physical systems are not completely understood, with a number of open questions remaining. Although mean-field random networks have certain limitations in modeling real-world systems, 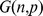-based network models have been employed in the literature to study various physical processes, including NMR sequential assignment [38], Potts glass formation [39], and relation of 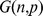 to quantum theory [40].

In general, according to our results, natural systems whose evolution can (to some extent) be described by a mean-field random network model would exhibit a first-order dense percolation transition, if the size 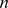 of the system is *very large* (e.g., a mole of liquid contains 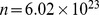 particles, which for most purposes can be considered 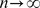). For instance, if one assumes that the process of bond formation in a large-scale system of particles has a purely probabilistic nature (i.e., indistinguishable particles moving randomly with respect to each other, allowing one to make a simplifying assumption that the formation of a link/bond between any two particles *occurs with the same probability*), then a “highly connected” cluster spanning the whole system (with the required edge density 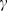, which may be set depending on characteristics of a particular system) would emerge abruptly when the probability of link formation 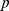 reaches 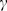. However, it should be noted that many real-life networks are not dense; therefore, examples of dense percolation transition phenomena with 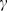 close to 1 cannot be easily identified in nature.

In addition, formula (1) has important implications in computational studies of the maximum 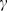-quasi-clique problem. As it turns out, identifying the largest 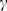-quasi-clique in a given network is an extremely computationally challenging task. The recent work [41] shows that this problem is NP-hard, which means that finding its exact solution for a network of size 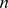 would require an exponential in 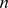 number of operations. The currently available exact methods allow one to explicitly compute the maximum 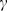-quasi-clique size only in small sparse graphs (*n*∼10^2^) [41]. Therefore, the performance of any inexact (approximate/heuristic) methods cannot be evaluated for *large-scale* networks. Thus, the theoretical bounds for the maximum 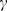-quasi-clique size in large-scale mean-field networks are of particular interest in the context of evaluating the performance of new computational algorithms that may be developed for this problem in future.

Overall, the results of this study provide a starting point for rigorous mathematical justifications for the existence of first-order phase transitions in large-scale linked systems. Despite the limitations of classical mean-field random networks, the process of network evolution under this model may capture certain aspects of the evolution of natural systems undergoing phase transitions. Moreover, due to the fact that dense percolation transition is already discontinuous without any changes to the mean-field model (as opposed to “regular” percolation that can be made discontinuous only after substantial modifications to the mean-field model), one can hypothesize that dense percolation may in fact be discontinuous for other network formation rules. Therefore, dense percolation transition can potentially be further analyzed in the context of other random network formation processes reflecting the behavior of physical, biological, and social systems.

## Materials and Methods

In this section we present graph-theoretic definitions and rigorous proofs of the aforementioned results. Since the nature of this study is theoretical rather than experimental, the main emphasis is put on mathematical proofs, rather than on data collection description.

Our formal arguments establish the existence of the first-order dense percolation transition in random graphs in the *asymptotic* (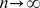) sense, i.e., for very large graphs. Note, however, that the mathematical techniques that support this result cannot be employed to draw conclusions on the situation with smaller networks. In view of that, we complement our theoretical findings by computational studies that illustrate the behavior of maximum 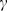-quasi-cliques in small- to moderately-sized graphs. Note also that the computational cost of conducting such experiments quickly becomes prohibitive as the size of the network increases: this is a direct consequence of the aforementioned fact that the maximum 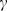-quasi-clique problem is NP-hard [41].

Due to the fact that the paper mainly considers the mean-field (Erdös-Rényi) random graph model, the networks utilized in numerical experiments were randomly generated using a straightforward procedure: for each value of 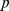, the link between each pair of nodes was generated randomly and independently with probability 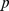.

### Graph-Theoretic Notations, Definitions, Related Work

A simple undirected graph 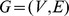 is defined in terms of its set of vertices (nodes) 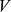 (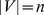) and its set of edges (links) 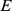. A *complete* subgraph, or a *clique* in a graph 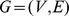 is a subset 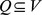 of vertices that are pairwise adjacent, i.e., for any 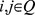 there exists an edge 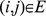. In the present work we are concerned with the properties and behavior of a certain type of *clique relaxation*, namely the 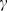 *-quasi-clique* [14].

**Definition 1** (γ-quasi-clique, or γ-dense subgraph) *Let 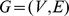 be a simple undirected graph, and 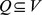 be a subset of its vertices. The (induced) subgraph 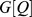 is called a 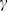
–quasi-clique for a given fixed 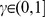, if the ratio of the number of edges in 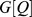 to the maximum possible number of edges among vertices in 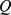 is at least* 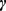:
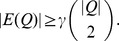



Note that the case of 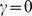 would be trivial, as any graph is a 0-quasi-clique. A complete graph is a 1-quasi-clique, hence 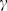-quasi-clique represents a *density*-based relaxation of the clique, as compared to *degree-* and *diameter-*based clique relaxations such as 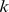-plex and 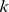-club [42]–[50]. A 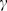-quasi-clique with the largest number of vertices is called the *maximum* 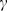-quasi-clique.

In this work, we investigate the asymptotic behavior of 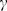-quasi-cliques in large-scale random graphs. In particular, we employ the well-known 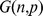 model of random graphs, originated by Erdös [51], which denotes a graph on 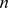 vertices, such that an edge between any two vertices exists with a probability 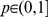, independently from other edges. Since the 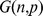 model yields graph instances with a rather “uniform” structure, as opposed to, for instance, *power-law* graphs, it is often called a *uniform random graph* model [52].

Random graphs and related structures, such maximum cliques in random graphs, have been studied intensively in last decades [24], [53], [54]. One of the earliest works on the asymptotic behavior of maximum clique in uniform random graphs is due to [55], who showed that the maximum clique size has a strong peak around 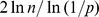. Grimmett and McDiarmid [25] proved that as 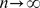 the maximum clique size in a uniform random graph 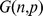 is equal to 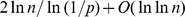 with probability one.

### Main Theorems and Remarks

First, we prove a generalization of the result of Grimmett and McDiarmid [25] for the case of maximum 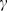-quasi-clique size. Furthermore, we demonstrate that the size of maximum 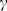-quasi-clique in 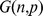 undergoes a *phase transition* when the value of 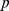 is varied in the vicinity of the (fixed) value 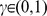, manifested in a sudden and drastic change of size of the maximum 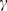-quasi-clique in 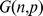 relative to the size of the graph itself. Specifically, in the next subsection we present the proofs of the following two theorems.

**Theorem 1** *If 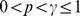, then the size 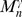 of the maximum 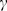-quasi-clique in a uniform random graph 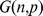 satisfies*

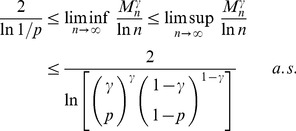
(3)


**Theorem 2** If 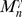 is the size of the maximum 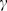-quasi-clique in a uniform random graph 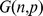 for some fixed 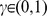, then with high probability (w.h.p.)
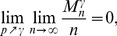
(4a)
*but*



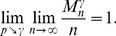
(4b)


Second, in addition to the computational experiments on relatively large graphs, where large 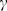-quasi-cliques were found using heuristic algorithms (due to NP-hardness of the maximum 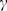-quasi-clique problem for any fixed 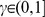, as stated above), we also conducted experiments on smaller graphs using a linear mixed integer programming formulation for the maximum quasi-clique problem, which allowed us to identify *exact* maximum quasi-cliques in graphs with up to 100 vertices. Interestingly, it turned out that the obtained asymptotic bounds were rather accurate for the considered small-scale uniform random graph instances. The details of these computational experiments are presented further in this section.

**Remark 1** Note that while in the context of this work we are interested in the largest *dense connected component*, the above definition of 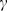-quasi-clique does not require connectivity. This allows for significant simplifications in the arguments that are presented below; however, the obtained results are still valid for the largest *connected* 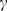-quasi-clique due to the following observations:

1. It can be easily shown that if 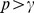, then the whole graph 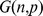 is w.h.p. a 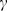-quasi-clique. Since under the model assumptions 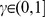 is a parameter that does not depend on the size of the graph 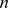, then if 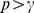, the whole graph 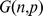 is automatically connected w.h.p., as a direct application of classical results by Erdös and Rényi [53]; therefore, the connectivity requirement for the largest 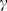-quasi-clique is w.h.p. satisfied in this case.
2. If the largest 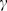-quasi-clique does not coincide with the whole graph 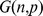 (this would correspond to the case 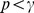), asymptotically precise upper and lower bounds (1) that will be proven for the size of the largest 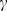-quasi-clique (in the context of Definition 1) are automatically valid for the largest *connected* 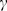-quasi-clique. This is due to the simple observation that the size of the largest connected 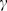-quasi-clique does not exceed the size of the largest (not necessarily connected) 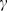-quasi-clique, and it is at least as large as the size of the maximum clique.

**Remark 2** The phase transition, a phenomenon of a drastic change in some property of a random structure over a small change in the structure’s parameters, is well known in the literature. With respect to random graphs, the limiting probability of a graph’s property changing from 0 to 1 or vice versa is well known for *monotone* and *first order* graph properties [56]. A property 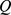 is *monotone increasing* (respectively, *decreasing*) if from 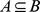 (resp., 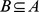) and 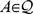 it follows that 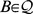. The first order graph properties are ones that can be finitely described in a first order language, i.e., language consisting of variables that represent graph vertices, equality 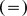 and adjacency (∼) relations, Boolean symbols 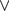, 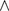, 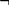, and the universal and existential quantifications 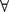, 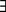. Note that first order properties are not necessarily monotone and vice versa; for instance, the increasing property “graph is connected” cannot be expressed in first order language [57]. Then, limiting relations similar to (23) that concern random graphs with first order properties 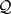 are known as *zero-one laws* [56], [57]:
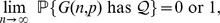
where the probability is monotone if 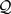 is monotone.

In this context, it is worth noting that the property that “graph is a 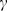-quasi-clique” in neither monotone, nor first order property, hence the phase transition in the relative size of 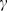-quasi-clique in uniform random graphs (23) *may not be obtained directly from the general zero-one laws relations*.

**Remark 3** Another related definition of the Erdös-Rényi random graph model that exists in the literature is 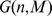 where graphs on 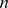 nodes with 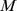 links are uniformly equiprobable with probability 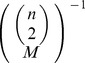. In this work, we adhere to the 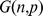 definition given above. All the presented results consider 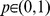 explicitly depending on 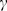 rather than on 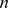 (that is, for sufficiently large 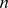, the condition 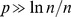 is always true), so the *whole graph* is with high probability (w.h.p.) connected according to the classical theory [53]. Although the largest connected component already coincides with the whole graph, the largest *dense connected component* may still be small compared to the size of the whole graph, which turns out to be the case for any 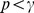.

**Remark 4** The obtained asymptotic bounds and first-order phase transition results are also valid for the size of the maximum 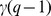 *-core* (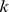-core with 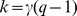 mentioned above), or, more generally, for the size of the largest subgraph that is required to have a certain minimum degree 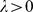 as a *percentage* of its size, in addition to the minimum edge density 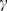. These subgraphs (clusters) are referred to as 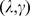-quasi-cliques [20]. Formally, a subgraph of 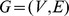 induced by the set of vertices 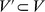 is a 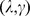-quasi-clique (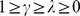) if and only if the following two conditions hold:
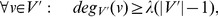
(5)

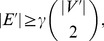
(6)where 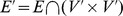 and 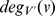 is the number of neighbors of vertex 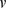 in set 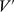.

Let 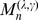 be the size of the maximum 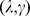-quasi-clique in random graph 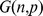. Observe that 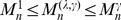, i.e., the maximum size of 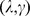-quasi-clique is always not greater than the maximum size of 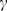-quasi-clique and bounded by the maximum clique size. It follows from the fact that any clique is a 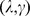-quasi-clique, and any 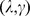-quasi-clique is a 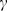-quasi-clique.

Therefore, the bounds in (21) are also valid for 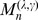. Moreover, it can be easily shown that when 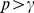


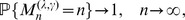
thus, the *first-order* phase transition in the point 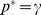 is also valid for the asymptotic behavior of the order of magnitude of the maximum 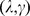-quasi-clique (including 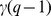-core as a special case).

### Rigorous Proofs of Main Results

Define 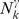 as the (random) number of 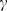-quasi-cliques of size 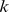 in 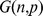. Noting that there are 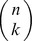 different subgraphs of size 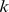 in this graph, let 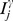 be the indicator variable such that 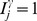 if 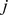-th subgraph of size 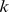 is a 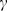-quasi-clique, 
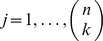
, and 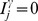 otherwise, which allows us to express 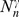 as
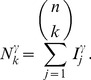
(7)


Obviously, the unconditional probabilities 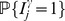 that any subgraph of size 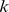 is a 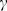-quasi-clique are equal, whence the expected number of 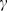-quasi-cliques of size 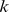 in 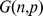 is given by
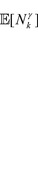
(8)where 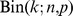 is the c.d.f. of the binomial distribution. As it will be seen, the integer 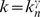 such that
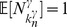
(9)for large values of 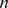 plays a central role in the sequel. The next proposition takes a first step in evaluating 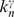.

**Proposition 1** *If 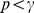, the integer 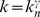 that satisfies* 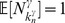 *increases with 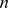 in such a way that 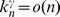, 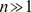
*.

*Proof.* From expression (8) it is evident that 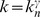 cannot be bounded for large values of 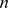, since in that case the right-hand side of (8) would be equal asymptotically to 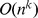. To verify that 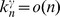, we construct an upper bound on the right hand side of equation (8). Using Stirling’s approximation, the binomial coefficient in (8) can be bounded as
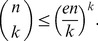
(10)To bound the summation term in (8), we use Chernoff’s bound for the tail of the binomial distribution [58]:
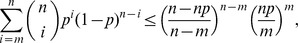
where 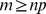. In our case 
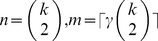
 (for simplicity, we use 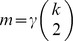), and since 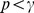, then 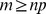; thus, the requirement on 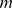 is valid. Thus,



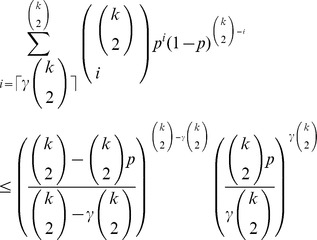


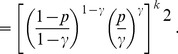
(11)


Combining the upper bounds in (10) and (11), we have that if 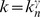 satisfies 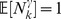, whereby the following must hold for large enough values of 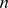:
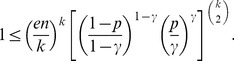
(12)


Taking logarithm of the right hand side of the above inequality and dividing by 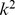, we obtain
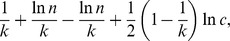
where the constant 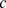 has the form 
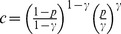
. It is easy to see from the inequality for arithmetic and geometric means that 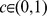 for 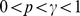. Then, if 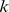 grows with 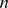 such that 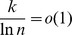, the above expression becomes negative for sufficiently large 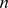, thereby contradicting the constructed upper bound (12). This implies that 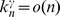, which proves the proposition.

**Proposition 2** *If 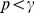, the integer 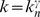 that satisfies the equality* 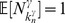, *is given by*

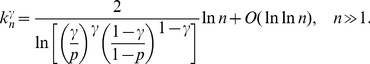
(13)


In establishing Proposition 2 we rely on the following result due to [59].

**Theorem 3** (McKay [59]). *Let 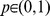 be fixed, and 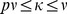 for some 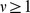. Define 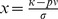, where 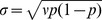. Then*

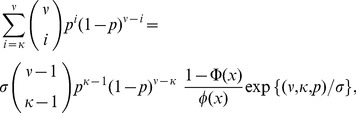
(14)where



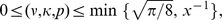

*and* 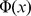 and 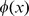 *are the cumulative and probability density functions of the standard normal distribution, respectively*.

*Proof of Proposition \reftheorem-1.* Using the notations of Theorem 1, let
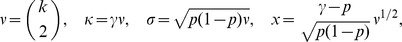
where we note that 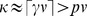 for large enough 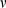, then the last term in (14) satisfies



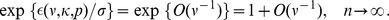



From the fact that 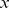 increases with 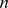 (cf. Proposition 1), it follows that
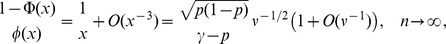
where the well-known expansion



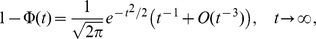
was used. Invoking Stirling’s expansion for 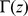,
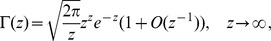
we obtain



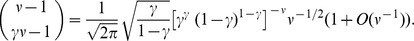



Thus, finally, the tail of the binomial distribution in (8) can be estimated as.
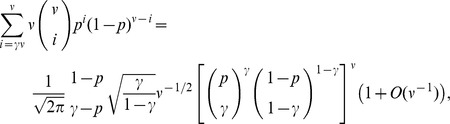
where 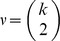. Consequently, equation (8) can asymptotically be written as



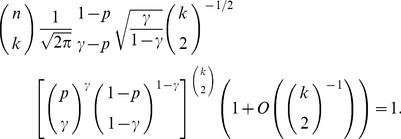
(15)Taking the logarithm of both sides of the last equality, we obtain
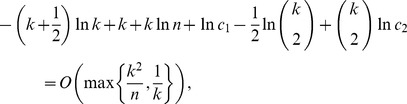
(16)where
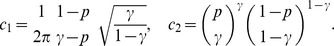



Taking into account that
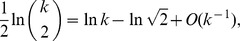

equation (16) can be written as



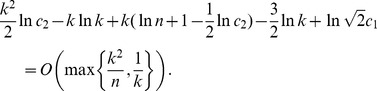



To obtain the main term of the asymptotical approximation of the solution of the last equation, let us restate it in the form.
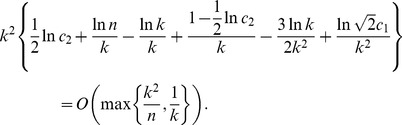



In view of the fact that 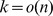 due to Proposition 1, the above expression can be further rewritten as
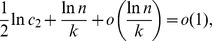
whence we have that



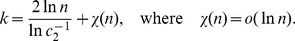



To determine the order of the term 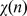, we restate the last equation as.
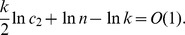



Writing down the expression for 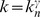 in the form.
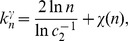
and substituting it in the last equation, we obtain that 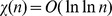, which furnishes the statement of the proposition.

Next, we demonstrate that the number 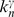 (given by Equation (13)), which solves the equation 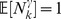, with probability 1 represents an upper bound on the size of the maximum 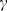-quasi-clique in a uniform random graph when 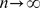. For this, we need the following property of 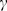-quasi-cliques.

**Proposition 3** *If graph 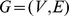, where 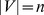, is a 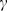-quasi-clique for some fixed* 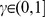, *then for any 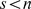 there exists a 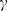-quasi-clique of size* 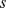 *in* 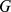.

*Proof.* For 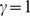, this property is trivial. In the case of 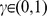, it suffices to show that the statement of the proposition holds for 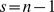. Since 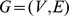 is a 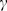-quasi-clique, then
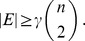



Assume that there exists a vertex 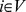 with 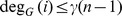. Let 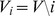; then the induced subgraph 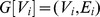 is also a 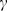-quasi-clique, since
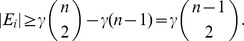



If there is no such a vertex, i.e., 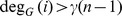 for all 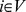, then let 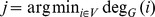 be the vertex of 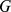 with the smallest degree, 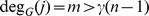. As before, denote 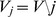, and observe that the cardinality of the set of edges 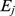 of the induced subgraph 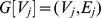 satisfies
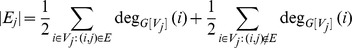


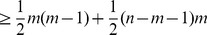


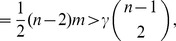
thus verifying the statement of the proposition.

**Proposition 4** *Let* 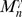 *denote the (random) size of the maximum 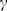-quasi-clique in a uniform random graph* 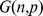. *If* 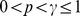, *then*

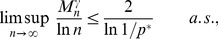
(17)

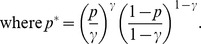



*Proof.* First, observe that
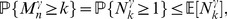
(18)where the equality is due to Proposition 3. Define a sequence 
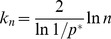
, 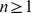; then, from expression (8) for 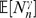, one obtains by following the steps in Proposition 2 that for sufficiently large values of 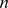




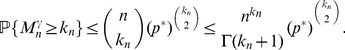
(19)From the definition of 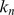 it follows that the term
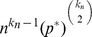
is bounded for large enough 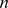, whence the sought probability can be subsequently bounded as



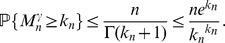



Again recalling the definition of 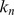, we note that
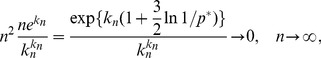
(20)which implies that 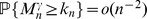 for 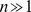, whereby the upper bound (17) on the size of the maximum 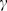-quasi-clique holds almost surely by virtue of the Borel-Cantelli lemma.

The next corollary shows that in sufficiently large random graphs 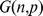, the size of the maximum 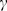-clique is almost surely above a certain value of the order of 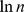. It uses a well known fact, established by [25], that the size of the maximum clique in a uniform random graph 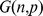 converges almost surely to 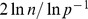. Note that 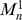 represents the size of the maximum clique (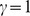) in a uniform random graph 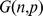. Observe also that, according to (13),
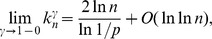
which corresponds to the well-known expression for the size of the maximum clique in uniform random graphs [24], [25]. This allows us to define 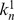 as the limiting value of 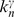 above.

**Corollary 1** *If 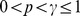, then the size* 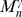 *of the maximum 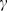-quasi-clique in a uniform random graph* 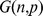 *satisfies*

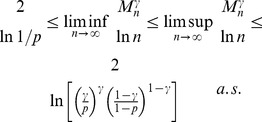
(21)where 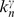 is given by (13).

*Proof.* This follows immediately from Proposition 4, and the observation that, for any 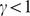, the size of the maximum 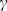-clique in 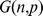 always greater than the size of the maximum clique in the same graph, i.e.,
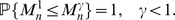



Also note that the relations 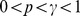 imply that
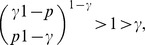
from which one infers the inequality



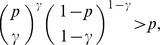
verifying that inequality for the lower and upper bounds on 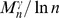 in (21) always holds, given the above assumptions on the values of 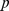 and 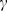.

**Remark 5** From Fatou’s lemma it follows that bounds (21) on the maximum 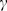-quasi-clique size 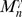, which hold with probability 1, also hold for the mean maximum 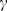-quasi-clique size, 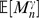:
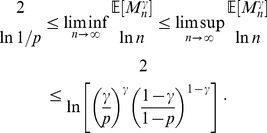
(22)


In such a way, we have established that for any fixed 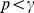 the asymptotic size of the maximum 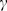-quasi-clique is of the order of 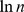. Intuitively, when 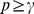, the entire graph 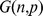 becomes a 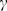-quasi-clique, thus the size of the maximum 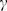-quasi-clique has the order of 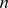. Therefore, the natural question arising here is what happens when 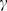 is fixed and 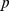 approaches 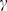. We show that there is a first-order phase transition in the asymptotic behavior of the order of magnitude of the maximum 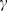-quasi-clique in the point 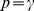.

**Proposition 5** *If* 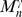 *is the size of the maximum* 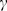 *-quasi-clique in a uniform random graph* 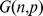 *for some fixed* 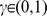 *, then with high probability (w.h.p.)*

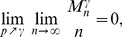
(23a)
*but*



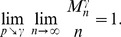
(23b)
*Proof.* The first limiting case follows from Proposition 4, since we proved that for any fixed 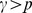 with probability 1
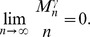



To prove the equality in (23b), let 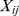 be a Bernoulli random variable which is equal to 1 if there exists an edge 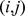 in the uniform random graph 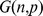. Then,
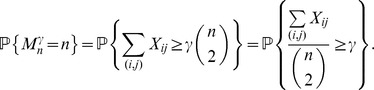



From the weak law of large numbers it follows that for any fixed 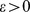,
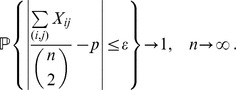



Letting 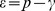, we obtain
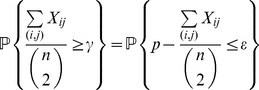


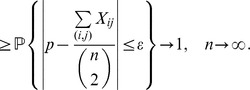



Therefore,
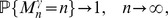
which ends the proof of the proposition.

### Linear Mixed-Integer Formulation of the Maximum 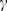-Quasi-Clique Problem

In this subsection we summarize a linear mixed-integer formulation of the maximum 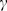-quasi-clique problem [41] that was used for “small-scale” computational experiments as a part of the Computational Experiments section below.

Consider a graph 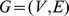 with 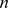 vertices and an adjacency matrix 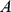 (with elements 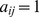 if there is an edge between vertices 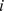 and 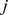, and 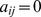 otherwise), and suppose that one selects some subgraph 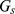 of 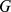. In order to verify whether 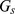 is a 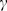-quasi-clique, we use the binary vector of variables 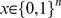, where 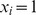 if vertex 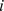 belongs to 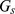, and 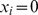 otherwise. The subgraph 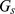 is a 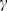-quasi-clique if the cardinality of its set of edges is at least
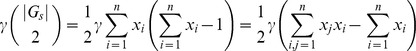


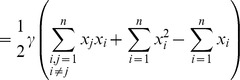


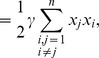
where the last equality is due to 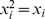. The number of edges in the subgraph 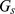 can be calculated as



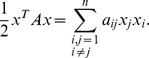



Therefore, the problem of finding the maximum 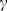-quasi-clique in the graph 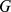 can be formulated as follows:
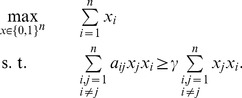
(24)


This is a 0–1 integer programming (IP) problem with a linear objective and a nonconvex quadratic constraint. A linearization of this problem can be performed at the expense of introducing additional variables and constraints.

A mixed-integer linear formulation of (24) with 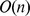 variables and constraints is given below. Recall that originally we had only one constraint,
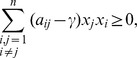
which can be rewritten as



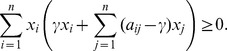



Define the variables 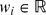, 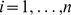, as follows
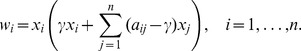



Next, observe that each of the quadratic equalities above is equivalent to four linear inequalities
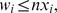


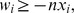


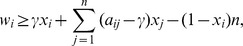


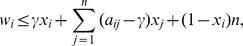


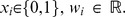



Therefore, the problem of finding a maximum 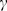-quasi-clique can be represented as the following mixed integer linear programming problem with 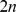 variables (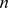 binary variables and 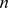 continuous variables) and 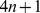 constraints. This formulation will be used for the computational experiments below.
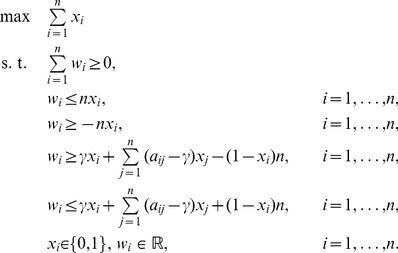
(25)


### Computational Experiments

In this subsection we consider exact and heuristic numerical computations of the size of the maximum 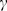-quasi-clique in randomly generated graphs. In particular, these computational experiments are aimed at checking the following two aspects: (i) how realistic the asymptotic bounds (21), (22) on the size of the maximum 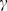-quasi-clique 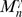 and its mean value are for relatively small values of 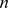 (*n*∼10^2^), and (ii) whether the approximate behavior of the relative size of the maximum 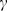-quasi-clique in *moderate-size* (*n*∼10^4^) random graphs (as 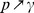) exhibits the “step function” pattern, which was proven for 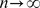 in Theorem 2.

According to Remark 5, in *large enough* random graphs 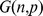 the average size 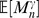 of the maximum 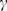-quasi-clique belongs to the interval
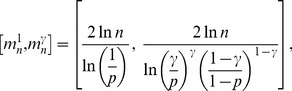
(26)provided that 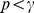. Therefore, it was of interest to check the applicability of the above bounds for relatively small values of 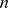.

To this end, in the first set of computational experiments we generated a number of instances of uniform random graphs 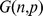 with 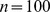 and 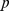 ranging from 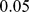 to 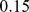; namely, we generated 100 instances of 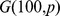 for every 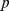. Then, we employed the MIP formulation (25) to find the maximum 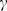-cliques in the generated graphs for values 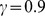 and 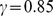. Such a choice of parameters is justified by relatively better numerical tractability of the MIP problem (25) for sparse graphs. We used FICO™ Xpress Optimization Suite 7.1 [60] to solve the resulting instances of problem (25). The resulting average values of 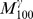, as well as the minimum and maximum values of 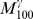 over 100 instances for each 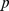, are reported in Table 1.

**Table 1:** Maximum 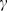-quasi-clique sizes in 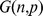 for 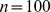, and 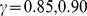.

	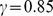			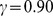		
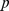	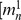 , 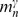 ]	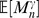	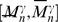	[ 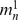 , 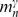 ]	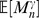	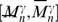
0.05	[3.07, 4.32]	3.10	[3], [4]	[3.07, 3.88]	3.10	[3], [4]
0.06	[3.27, 4.66]	3.26	[3], [5]	[3.27, 4.16]	3.26	[3], [5]
0.07	[3.46, 4.98]	3.38	[3], [5]	[3.46, 4.44]	3.38	[3], [5]
0.08	[3.65, 5.30]	3.60	[3], [5]	[3.65, 4.71]	3.60	[3], [5]
0.09	[3.82, 5.62]	3.97	[3], [5]	[3.82, 4.97]	4.97	[3], [5]
0.10	[4.00, 5.94]	4.27	[3], [5]	[4.00, 5.50]	4.24	[3], [5]
0.11	[4.17, 6.26]	4.66	[4], [5]	[4.17, 5.77]	4.65	[4], [5]
0.12	[4.34, 6.58]	4.87	[4], [6]	[4.34, 6.04]	4.82	[4], [5]
0.13	[4.51, 6.91]	5.06	[5], [6]	[4.51, 6.04]	5.00	[5,5]
0.14	[4.68, 7.25]	5.19	[5], [6]	[4.68, 6.31]	5.01	[5], [6]
0.15	[4.85, 7.59]	5.67	[5], [7]	[4.85, 6.59]	5.06	[5], [6]

Average (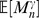), minimum (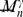), and maximum (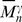) observed values of the largest 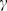-quasi-clique size, computed for 100 instances of uniform random graphs 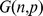 for 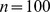 and 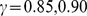. Theoretical lower and upper bounds [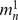, 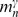] are calculated using formula (29).

**Figure 2:**
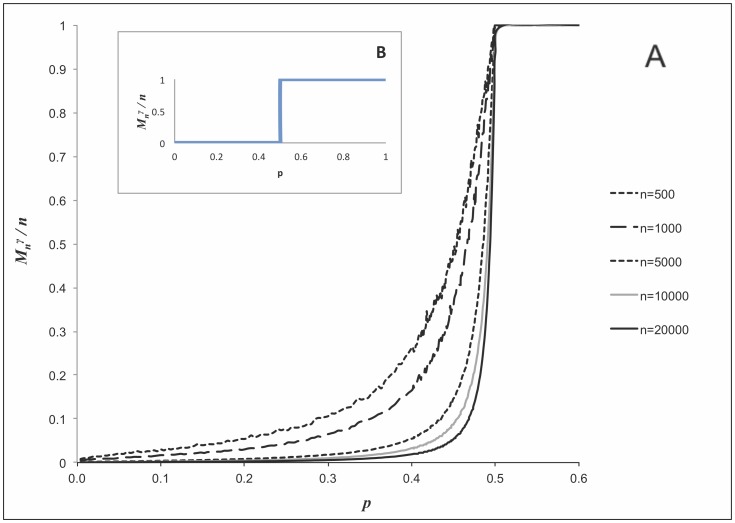
Illustration of the behavior of the largest dense cluster size in moderate-size graphs 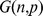. (**A**) Relative size of maximum 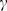-quasi-cliques (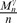) in random graphs 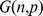 for 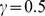 (this particular value is chosen simply for illustrative purposes: the obtained results hold for any fixed 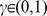) and 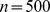, 1,000, 5,000, 10,000, 20,000. Due to the fact that finding the maximum quasi-clique in a graph is a computationally challenging NP-hard problem (as opposed to finding the largest “regular” connected component, which can be done in polynomial time), the numerical simulations were carried out using GRASP heuristic algorithm [14] and plotting the largest relative size found after multiple runs of the algorithm for each 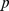. The growth increment of 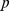 was chosen at 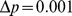 in the region where 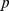 approaches 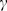 from below (more details on computational experiments are given in Materials and Methods). (**B**) *Theoretical* behavior of the relative size of the maximum 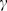-quasi-clique in 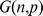 as 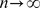, which is simply a step function, as indicated by formulas (2a)–(2b).

**Figure 3:**
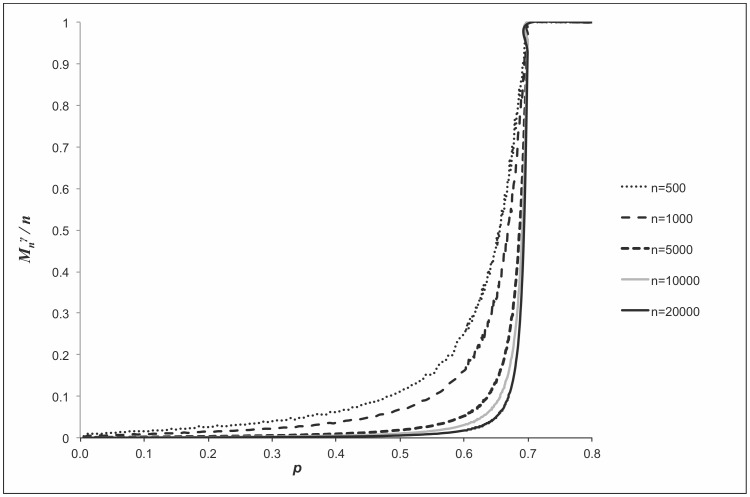
Illustration of the behavior of the largest dense cluster size in moderate-size graphs 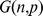. Relative size of the maximum 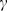-quasi-cliques in the uniform random graphs for 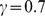, 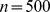, 1,000, 5,000, 10,000, 20,000, and 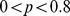. All notations are the same as in the caption of Fig. 2.

In the second set of computational experiments, we analyzed the behavior of the relative size of the maximum 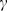-quasi-clique for a fixed 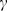 and different values of 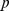 and 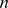. For 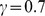, two sequences of values of parameter 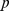 were defined: 
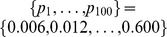
 and 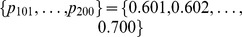. Note the smaller “step size” of the second sequence, which allows for a more thorough investigation of the maximum 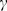-clique size when the value of 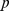 becomes close to 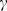. The same experiment setup (with an appropriate adjustment of the sequence of values of 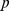) was used for 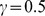. For each value of 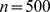, 1,000, 5,000, 10,000, 20,000 and 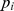 from the sequence defined above, we generated instances of uniform random graphs 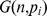, and determined the maximum 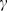-quasi-clique size 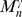. Since the MIP formulation (25) becomes computationally intractable for large dense graphs, we used maximum 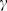-quasi-clique GRASP heuristics due to [14], which were reported to perform quite well in massive graphs. Figures 2 and 3 report the results of the described computational experiments.

Recall that it was shown in above that with probability that approaches to 1 it holds that
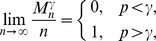
or, in other words, the relative size of the maximum 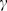-quasi-clique in uniform random graphs as a function of the density 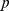 of the graph with high probability represents a step function as 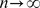. The results presented in Figures 2 and 3 complement these theoretical findings and show the respective behavior for “medium-scale” random graph instances.
